# Assessment of Two Types of Headphones Used in a Tablet-Based Automated Hearing Screening Method in Different Noise Conditions

**DOI:** 10.1055/s-0045-1811515

**Published:** 2026-03-11

**Authors:** Camila P. Aquino, Clayton H. Rocha, Camila M. Rabelo, Seisse Gabriela Gandolfi Sanches, Carla G. Matas, Alessandra G. Samelli

**Affiliations:** 1 Department of Physical Therapy, Speech-Language-Hearing Sciences, and Occupational Therapy, Faculty of Medicine, Universidade de São Paulo (FMUSP), São Paulo, SP, Brazil

**Keywords:** hearing loss, audiology, noise, internet, audiometry

## Abstract

**Objective:**

To compare the performance of two types of supra-aural headphones in both silence and noise in automated hearing screening in adults via tablet computers.

**Methods:**

Basic audiological assessments and audiometry screenings via tablet were conducted in silence and noise in 35 participants. The hearing screening application assesses frequencies of 1, 2, and 4 kHz (20 dBHL) and 0.5 kHz (30 dBHL) in each ear separately. Two different headphones were tested: TDH 39 (Telephonics Corporation) and Sennheiser HD 280 PRO (Sennheiser) (with passive noise cancellation)—in silence, white noise, and cafeteria noise, emitted in a free field. The screening results were compared with the gold standard (audiometry).

**Results:**

The results of the 35 participants with Sennheiser HD 280 PRO were compatible with the gold standard in the 3 situations. With TDH 39, the results of 33 individuals in silence and 34 in white noise were compatible with the gold standard. Both headphones, in automated screening in silence and noise, had 100% sensitivity, more than 93% specificity, and more than 94% accuracy.

**Conclusion:**

Both headphones performed well in the tablet automated hearing screening in the three situations. The HD 280 PRO performed better in silence and noise regarding specificity and accuracy. In cafeteria noise, there was no difference between the two headphones.

## Introduction

Deafness is a health problem that affects more than 5% of the world population, that is, 466 million people. It is estimated that by 2050 more than 900 million people—1 in every 10—will have disabling hearing loss. 1

Hence, primary hearing health care measures must include early promotion, prevention, and identification of hearing problems in the community, as well as referrals to mid- and high-complexity services, when necessary. 2 However, these measures are not enough to meet or even identify the needs, further aggravated by the limited number of hearing health professionals 3 and sometimes precarious hearing health services. 4

Hearing loss is diagnosed with a basic hearing evaluation, including pure-tone audiometry (PTA), speech tests and immittance acoustic measurements, using clinical audiometer, which further limits the access of inhabitants of remote areas to this assessment. 5

According to the World Health Organization (WHO), 6 telemedicine encompasses health care services provided when distance is a critical factor. Teleaudiology is a more recent specific field in which telemedicine is used, applying its principles to audiology practice. 3

Tele-audiometry, one of the uses of teleaudiology, automatically simulates audiometry tools and patterns with software installed in a computer. It provides patients living in remote areas the possibility of access to diagnostic services, decreasing the costs to patients, the government, and professionals who screen them, as no specific and complex equipment is required. 5 7

The importance of teleaudiology grew during the current coronavirus disease 2019 (COVID-19) pandemic. 8 It recently caused extensive interruptions in health services worldwide, consequently hastening the need for a remote hearing health service model. 9 10

Two studies have demonstrated the effectiveness of using automated audiometry applications as an accessible, simple, and early way to detect hearing loss in the population, especially children. 7 11

However, environmental noise in hearing screening settings (since schools are normally noisy settings) was a difficulty commonly found in such studies. 8 12 13 Even though the noise is monitored, such hearing assessments are impaired, especially at lower frequencies, like 0.5 kHz. 11 13

A recent study suggested the need for increasing stimulus intensity at 1 kHz in screening because of environmental noise. Thus, the influence of noise on the results and the referral rates due to screening failures would be minimized. 14

Therefore, based on the difficulties found in hearing screening in noisy environments and aiming for solutions to minimize noise interference with hearing assessments, the objective of the present study was to compare the performance of two types of supra-aural headphones in silent and noisy situations, in adults, during automatic hearing screening, carried out using a tablet.

## Methods

Adults attended at the Audiology Service of the University Hospital of Universidade de São Paulo were invited to participate. The convenience sample consisted of volunteers based on the following inclusion criteria: age between 18 and 50 years and understanding the instructions for carrying out pure tone audiometry (PTA), as well as hearing screening using a tablet. All individuals who met the inclusion criteria, regardless of whether they had normal hearing thresholds or not, were included. The presence of hearing complaints was not an impediment to inclusion in the sample, due to the nature of the study. Thus, 35 adults participated in the study, 22 women and 13 men.

The following procedures were used:

- Otoscopy and acoustic immittance.
- PTA to determine air-conduction hearing thresholds at 0.25, 0.50, 1, 2, 3, 4, 6, and 8 kHz in both ears, using TDH-39 headphones (Telephonics Corporation) in a sound booth. In case these thresholds were higher than or equal to 20 dBHL (0.50–4 kHz), bone-conduction thresholds were also evaluated with a bone vibrator at these same frequencies. Hearing thresholds higher than 20 dBHL were considered hearing loss. 15 In such cases, the hearing loss was classified by type (conductive, sensorineural, or mixed) 16 and degree (21–40 dBHL: mild; 41–70 dBHL: moderate; 71–90 dBHL: severe; > 91 dBHL: profound, based on the mean thresholds at 0.5, 1, 2, and 4 kHz). 17
- Automated hearing screening: conducted with tablet computers, using the P.E.T.I.T. application 7 and headphones. This application was developed and validated 18 to simulate tablet automated hearing screening, with a remote connection interface and a central data bank over the internet. The algorithm in this application assesses the 1, 2, and 4 kHz frequencies at 20 dBHL and 0.5 kHz at 30 dBHL. Stimulation is made with 2.5-second warble tones. The system was calibrated according to the following norms: ISO-8253-1-1989, 19 ISO-8253-2-1992, 20 ISO-389-1-1998, 21 IEC-60318-1-1998, 22 and IEC-60318-3-1998. 23 All frequencies and intensities produced by the iPad (Apple Inc.) in both headphones were within acceptable limits, according to ANSI S3.6-1996. 24

After assessing all frequencies in both ears, the application automatically gives the positive or negative screening result. It also automatically saves the data, which are stored in the iPad until they are sent via Wi-Fi to the central data bank for result assessment and management. 18

To pass the screening, subjects must respond to at least 2 of the 3 tones emitted 12 15 at 20 dBHL at each of the frequencies 1k, 2k, and 4 kHz and at 30 dBHL for the frequency of 0.5 kHz, in both ears. If they fail in one ear or frequency, the application automatically classifies the result as “fail”.

In the present study, the hearing screening (with tablet) was conducted with 2 headphones, namely: TDH 39 (the same professional headphones used to validate the application) 18 and Sennheiser HD 280 PRO (Sennheiser) (with passive noise cancellation [PNC]). Of the participants, 17 began screening with TDH, and 18 with HD 280 PRO headphones, to prevent this variable from influencing the results.

The first stage of the screening for all participants, regardless of the headphone they used first, was in silence in a sound booth. Afterward, the screening was conducted in noise, in a free field—first white noise and then cafeteria noise, presented at 50 dB(A) in a sound booth. The loudspeaker was positioned 100 cm away from the participant, at 0° azimuth. After the three screenings with the first headphone, the whole process was repeated with the second one.

The results of each screening were compared with the gold standard, that is, PTA conducted in a sound booth. Screening and audiometry were performed by different audiologists, depending on service availability.

Descriptive statistical analyses were made, and the diagnostic values of the screenings in each situation were calculated.

Individuals were divided into two groups—pass and fail—based on screening results and previously described analysis criteria. These groups were then compared with normal and abnormal results in PTA. Based on these comparisons, the following measures and respective 95% confidence intervals were calculated: 25 sensitivity, specificity, positive predictive value (PPV), negative predictive value (NPV), positive likelihood ratio, and accuracy. Statistical analyses were made in MedCalc, version 17.0.4 for Windows (MedCalc Software Ltd.).

## Results

Regarding sex and age, 22 subjects were female (62.85%), with a mean age of 26.27 years ±  4.68, and 13 were male (37.15%), with a mean age of 25.3 years ±  7.53. The minimum age was 18 years, and the maximum was 49 years.

The mean hearing thresholds obtained with PTA in a sound booth are shown in Table 1 . Of the 35 individuals, 2 (5.8%) had hearing loss (one had unilateral mild-to-severe hearing loss, and the other had bilateral moderate hearing loss); the other 33 (94.2%) had normal hearing. As for type, the unilateral hearing loss was mixed, and the bilateral was sensorineural.

**Table 1:** Mean and standard deviation of hearing thresholds at 0.5, 1, 2, and 4 kHz in PTA

	Right ear				Left ear			
Frequency (in kHz)	0.5	1	2	4	0.5	1	2	4
Mean (in dBHL)	7.85	5.42	4.14	5.42	7.85	6.42	4	8.42
Standard deviation	3.88	3.71	4.77	8.85	5.32	6.36	5.53	15.3
Minimum	5	0	0	0	0	0	0	0
Maximum	20	15	20	50	30	35	25	80

Screening without noise had two false-positive results with TDH 39. Sensitivity was 100% with both headphones ( Table 2 ).

**Table 2:** Sensitivity, specificity, positive predictive value, negative predictive value, positive likelihood ratio, and accuracy in all situations assessed in the study, considering PTA as the gold standard

Conditions	Se (95% CI)	Sp (95% CI)	PPV (95% CI)	NPV (95% CI)	PLR (95% CI)	A (95% CI)
**Silence** **(TDH headphone)**	100% (15.81–100.00%)	93.94% (79.77–99.26%)	50% (20.70–79.30%)	100%	16.50 (4.31–63.22)	94.29% (80.84–99.30%)
**Silence** **(HD 280 headphone)**	100% (15.81–100.00%)	100% (89.42–100.00%)	100%	100%	—	100% (90.00–100.00%)
**White noise** **(TDH headphone)**	100% (15.81–100.00%)	97.06% (84.67–99.93%)	66.67% (22.48–93.24%)	100%	34 (4.93–234.47)	97.22% (85.47–99.93%)
**White noise** **(HD 280 headphone)**	100% (15.81–100.00%)	100% (89.42–100.00%)	100%	100%	—	100% (90.00–100.00%)
**Cafeteria noise** **(TDH headphone)**	100% (15.81–100.00%)	100% (89.42–100.00%)	100%	100%	—	100% (90.00–100.00%)
**Cafeteria noise** **(HD 280 e headphone)**	100% (15.81–100.00%)	100% (89.42–100.00%)	100%	100%	—	100% (90.00–100.00%)

**Abbreviations:** A, accuracy; CI, confidence interval; NPV, negative predictive value; PLR, positive likelihood ratio; PPV, positive predictive value; Se, sensitivity; Sp, specificity.

Screening with white noise obtained 100% sensitivity with both headphones ( Table 2 ). Likewise, there were false-positive results with TDH 39.

Screening with cafeteria noise obtained 100% sensitivity and specificity with both headphones ( Table 2 ).

As for accuracy in both silence and white noise, HD 280 headphones had better results, although diagnostic values were high with both headphones ( Table 2 ).

## Discussion

The objective of the current study was to compare the performance of 2 types of supra-aural headphones—TDH 39 (traditional) and HD 280 PRO (with PNC)—in tablet automated hearing screening in adults in both silence and noise.

Even though the screening in question was designed for schoolchildren, this study applied it to adults. The assessment using both headphones in silence and noise requires the subjects' attention for a long time, as the whole process takes about 60 minutes. Hence, like other studies in the field, the headphones were initially assessed in adults to be later validated in children.

### Prevalence of Hearing Loss

The mean hearing thresholds obtained with pure-tone audiometry were normal at 0.5, 1, 2, and 4 kHz in both ears. However, 2 out of the 35 participants had hearing loss. Thus, the prevalence of hearing loss in this study was 5.7%. This value is lower than in previous studies (13% and 20% prevalence) 7 26 that also assessed adults with tele-audiometry. Such a difference demonstrates the importance of larger samples, with more participants with different types and degrees of hearing loss, to verify whether it would influence the results.

### Sensitivity, Specificity, Predictive Value, and Accuracy

TDH 39, the headphones traditionally used in hearing screenings and PTA, in silence, had 100% sensitivity, 93.94% specificity, and 94.29% accuracy. Thus, its performance in automated hearing screening was good, a result that agrees with the previous studies 7 18 that validated the P.E.T.I.T. application using TDH 39.

Yeung et al. 5 conducted a study with 70 children aged 3 to 13 years, using the ShoeBOX tablet application (WSA, Canada), which automatically assesses the frequencies from 0.5 to 4 kHz. The assessment was made in silence with TDH 39, in a sound booth, at a hospital audiology clinic. The hearing loss criterion was 25 dBHL. They found 93.3% sensitivity, 94.5% specificity, 98.1% NPV, and a 17.1 positive likelihood ratio.

Ferrari et al. 27 compared the results of hearing screening with a portable audiometer connected to those of a computer and with a conventional audiometer, using TDH 39. Both collections were made in a sound booth in silence, in two different groups. Group A comprised 30 adults, aged 18 to 41 years, with no hearing complaint, while group B comprised 30 adults, aged 23 to 85 years, all of them with hearing complaints. The test with a conventional audiometer and TDH 39 had 91.1% sensitivity, 94% specificity, 96.6% PPV, and 84.8% NPV. Therefore, the diagnostic values found in the present study are similar to those obtained in the previous ones, 5 7 18 27 demonstrating the effectiveness of using TDH 39 in silence.

In the present study, the results of 31 out of the 33 normal-hearing participants in automated hearing screening in silence with TDH 39 agreed with those of the gold-standard pure-tone audiometry. On the other hand, even though the hearing screening was conducted in silence in a sound booth, two adults had false-positive results, that is, they failed the screening, despite their normal hearing. One hypothesis for this result is the occlusion effect produced by the headphone. A previous study has described that supra-aural headphones can produce a greater occlusion effect than circumaural headphones. 28

Despite this result, the NPV was 100%, that is, participants who pass the hearing screening with TDH 39 are 100% likely to have normal hearing.

The results of both individuals with hearing loss were compatible with those of the gold-standard pure-tone audiometry, using TDH 39, that is, 100% sensitivity. Although there were few individuals with hearing loss, the sensitivity of the instrument with TDH 39 has already been approached in previous studies in children (95.7% sensitivity 18 ) and adults (100% sensitivity 7 ) with larger samples (244 children and 30 adults) and higher prevalence of hearing loss in both cases (respectively 9.43% and 13.3%).

Automated hearing screening with HD 280 PRO (headphones with PNC) in silence resulted in 100% sensitivity, specificity, and accuracy, that is, it proved to be an efficient transducer in automated screening.

Hussein et al. 29 also used HD 280 PRO in automated hearing screening with the hearScreen application (hearX, South Africa). They screened 6,424 children aged 3 to 6 years in a silent but not acoustically treated setting. The HD 280 PRO performed well in this study, although the authors highlighted the need for monitoring external noise during the assessment.

As for the present study, the NPV was 100%, which means that individuals who pass automated screening with HD 280 PRO in silence are 100% likely to have normal hearing. Likewise, since the PPV was also 100%, individuals who fail automated hearing screening with HD 280 PRO in silence are 100% likely to have auditory changes.

The current study corroborated the findings of previous ones, in which headphones with PNC proved to be efficient in automated hearing screening in silence, even when not conducted in an acoustically treated sound booth, 29 30 if they are duly calibrated for the operating system in which they will be used. 30

Noise can negatively interfere with hearing screening results. 11 12 13 Some studies have even resorted to a screening application tool that pauses the screening when the noise gets louder than the maximum permissible level. 30

The P.E.T.I.T. application has been validated upon constantly monitoring noise, which ensures reliable final results. 7 18

To investigate this problem and enable hearing screening at schools with as little interference from noise as possible, the current study sought to identify which headphones would be less influenced by it. Thus, the maximum permissible environmental noise level 50 dB(A)—for screenings at schools was simulated in a sound booth, 31 using both white noise and cafeteria noise.

White noise was used in this study because it is a broadband, rather encompassing noise. 32 Cafeteria noise was used for having a speech spectrum and amplitude modulations, simulating everyday interference situations. 33

The TDH 39 performed well in automated hearing screening in both white noise and silence. It had 100% sensitivity, 97.06%, specificity, and 97.22% accuracy.

The PPV was 66.67%, that is, individuals who fail the screening with TDH 39 in noise are 66.67% likely to have auditory changes. The NPV was 100%, which means that individuals who pass the screening are 100% likely to have normal hearing. Screening and PTA results were 100% compatible regarding true positives (sensitivity), but one normal-hearing subject failed the screening with TDH 39. A hypothesis for this incompatible result is less noise cancellation, which is to be expected from supra-aural headphones in comparison with circumaural ones. 28

In the same above situation, the HD 280 PRO performed well in white noise, with 100% sensitivity, specificity, accuracy, PPV, and NPV. These values reinforce the feasibility of using HD 280 PRO in automated hearing screening, even in acoustically untreated settings. 29 30

In cafeteria noise, both TDH 39 and HD 280 PRO headphones performed well and had equal diagnostic values—100% sensitivity, specificity, accuracy, PPV, and NPV. Since speech frequencies predominate in this noise spectrum and the screening uses warble stimuli, a hypothesis for this result is that noise interference was not quite as decisive as in white noise. Moreover, in this testing condition, both types of headphones provided adequate cancellation, ensuring good performance.

As limitations of the study, we can highlight the sample size, as already mentioned. It is known that the predictive values of a screening depend on the prevalence of the disease in the population tested. 34 In the case of the present study, we found a prevalence of hearing loss of almost 6%, which is a prevalence similar to that estimated by WHO (2021) for the global population with degrees of hearing loss above moderate. 35 However, as our sample is small, such results do not allow generalization. Nonetheless, it is important to highlight that the predictive values observed in the study were, in general, high. In addition, we are already replicating this study in a pediatric sample, which is the target population, and in a real environment, which will provide additional data regarding the use of screening with noise-canceling headphones in a school environment.

The strength of the present study is the evaluation of two headphones (one traditional—TDH 39—of higher cost, and another commercial, with passive noise cancellation—Sennheiser HD 280 PRO—and of lower cost), carried out in a controlled environment, with different acoustic conditions presented in a randomized fashion (silence and different types of noise), whose results were compared to the gold standard test, providing a robust methodology for verifying the diagnostic performance in each of the conditions, before this tool is used in a real world condition.

## Conclusion

The findings of this study revealed that both TDH 39 (traditional headphones) and HD 280 PRO (headphones with PNC) performed well in tablet automated adult hearing screening, in both silence and noise. The diagnostic hearing screening values were high with both headphones—100% sensitivity, more than 93% specificity, and more than 94% accuracy. The HD 280 PRO performed better in silence and white noise regarding specificity and accuracy. In cafeteria noise, there was no difference between the two headphones.
